# Lycodine-Type *Lycopodium* Alkaloids from the Whole Plants of *Huperzia serrata*

**DOI:** 10.1007/s13659-017-0140-z

**Published:** 2017-07-25

**Authors:** Yu-Chen Liu, Zhi-Jun Zhang, Jia Su, Li-Yan Peng, Lu-Tai Pan, Xing-De Wu, Qin-Shi Zhao

**Affiliations:** 1 State Key Laboratory of Phytochemistry and Plant Resources in West China, Kunming Institute of Botany, Chinese Academy of Sciences, Kunming, 650201 People’s Republic of China; 2 Guiyang College of Traditional Chinese Medicine, Guiyang, People’s Republic of China; 3 University of Chinese Academy of Sciences, Beijing, 100039 People’s Republic of China

**Keywords:** *Lycopodium* alkaloids, Lycodine-type, *Huperzia serrata*, BACE1 inhibitory activity

## Abstract

**Abstract:**

Three new lycodine-type *Lycopodium* alkaloids, namely 1-methyllycodine (**1**), 8*α*-hydroxy-15,16-dehydro-des-*N*-methyl-*α*-obscurine (**2**), *N*-methyl-16-hydroxyhuperzine B (**3**), and one new natural lycodine-type *Lycopodium* alkaloid, *N*-methylhuperzine A (**4**), along with 11 known analogues (**5**–**15**), were isolated from the whole plants of club moss *Huperzia serrata*. The structures of **1**–**4** were elucidated on the basis of NMR spectroscopic and mass spectrometry data. Among them, compound **1** was the first lycodine-type alkaloid possessing a methyl group at C-1. In addition, the structure of **5** was confirmed by the single-crystal X-ray crystallography data and its ^13^C NMR was reported for the first time in current study. Compounds **1**–**5** were tested their BACE1 inhibitory activity.

**Graphical Abstract:**

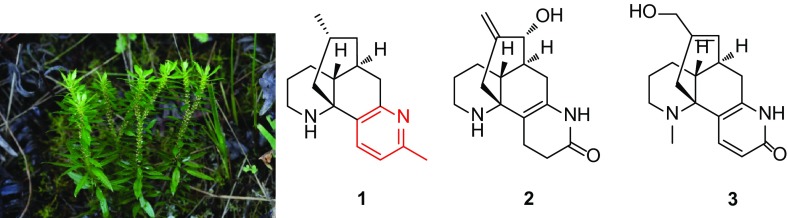

**Electronic supplementary material:**

The online version of this article (doi:10.1007/s13659-017-0140-z) contains supplementary material, which is available to authorized users.

## Introduction

The *Lycopodium* alkaloids, which only found from the plants of the families Lycopodiaceae and Huperziaceae, have attracted broad interest from chemists and pharmacologists worldwide due to their intriguing carbon skeletons and biological activities [1–6]. Such alkaloids were divided into four major classes (lycopodine, lycodine, fawcettimine, and phlegmarine) by Canadian famous chemists Ayer et al. [7]. Particularly, lycodine-type alkaloids, which generally characterized by four connected six-membered rings, including a pyridone or pyridine ring, a piperidine ring, and a bicyclo[3.3.1]nonane core [8], are a unique class of compounds and have attracted great interest for their biological activities especially the extraordinary acetylcholinesterase (AChE) inhibition by huperzine A that has a potential of becoming a therapeutic agent for the treatment of Alzheimer’s disease [2, 9].

**Fig. 1:**
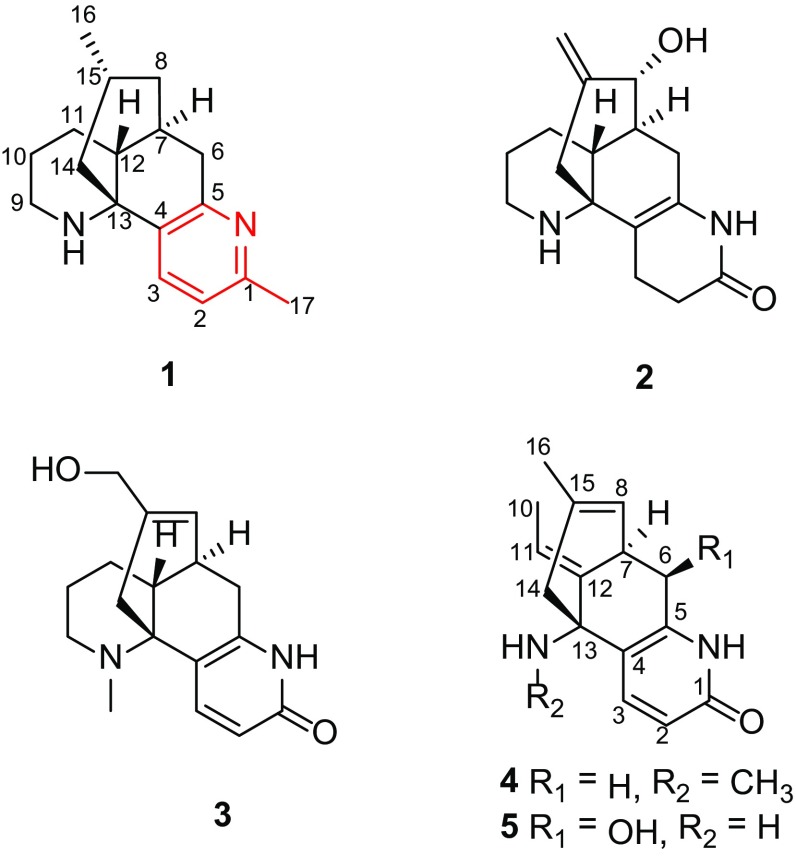
Structures of compounds **1**–**5**

**Fig. 2:**
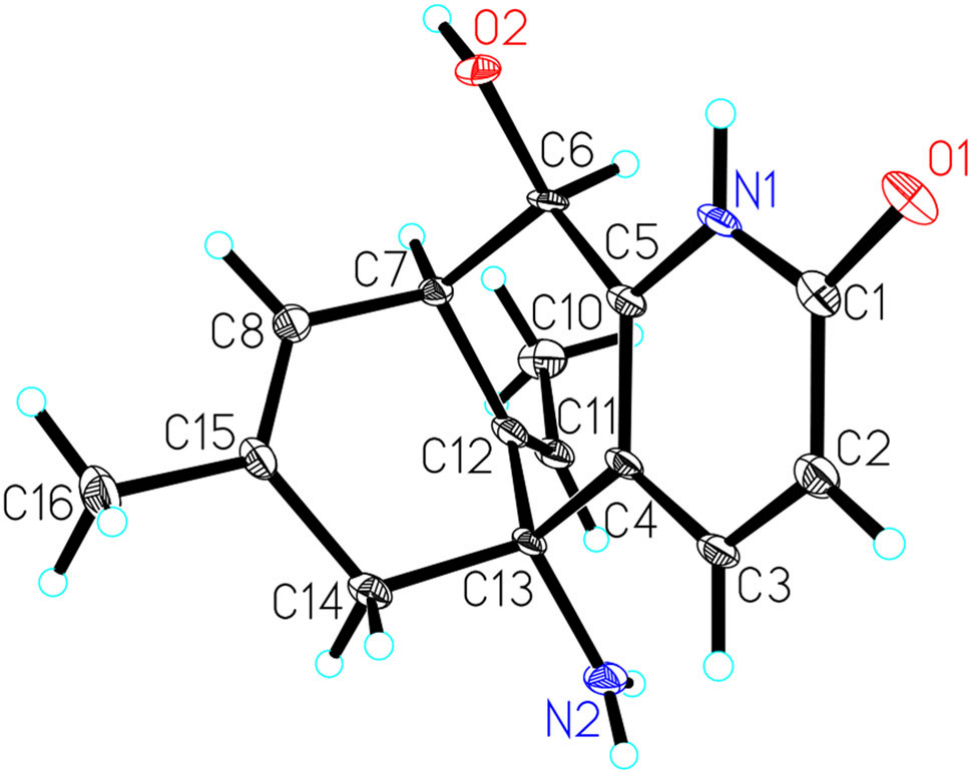
The X-ray structure of compound **5**

Chinese traditional medicinal plant *Huperzia serrata*, chiefly growing in rock crevice or somewhere dank in the forests, shrubs, or roadsides at the elevation of 300–2700 m in Southwestern and Southeastern China, belonged to the Huperziaceae family and was known for its therapeutic effect on contusions, pains, swellings, schizophrenia, and organophosphate poisoning in ancient China [10, 11]. Previously, phytochemical studies on *H. serrata* have led to a series of components [12, 13]. Among them, lycodine-type *Lycopodium* alkaloids were its main chemical and bioactive ingredients [14, 15], in particular, a type of alkaloids that possessed extraordinary AChE inhibition, such as the well-known huperzine A [2, 9]. As part of an ongoing research program aimed at exploring more *Lycopodium* alkaloids with fascinating structures and bioactivities serving as lead compounds for drug discovery, three new lycodine-type alkaloids, 1-methyllycodine (**1**), 8*α*-hydroxy-15,16-dehydro-des-*N*-methyl-*α*-obscurine (**2**), *N*-methyl-16-hydroxyhuperzine B (**3**), and one new natural *Lycopodium* alkaloid *N*-methylhuperzine A (**4**) were isolated from *H. serrata* (Fig. 1), together with 11 known analogues. The known compounds were identified as 6*β*-hydroxyhuperzine A (**5**) [16], huperzine A (**6**) [2, 9], huperzine B (**7**) [2, 9], casuarine B (**8**) [17], huperzinine (**9**) [18], lycodine (**10**) [19], *N*-methyllycodine (**11**) [20, 21], carinatumine B (**12**) [22], des-*N*-methyl-*β*-obscurine (**13**) [23], *N*-demethylhuperzinine (**14**) [24], and 16-hydroxyhuperzine B (**15**) [25] by comparison of their spectroscopic data with those reported in the literature. Their structures were determined by extensive spectroscopic analysis. Among them, the structure of **5** was confirmed by the single-crystal X-ray crystallography data (Fig. 2) and its ^13^C NMR data (Table 2) was reported for the first time in current study. In addition, these findings might provide more information for the biological activities study and synthesis of lycodine-type alkaloids. Reported herein are the isolation, structure elucidation and bioactivity investigation results of compounds **1**–**5**.

## Results and Discussion

The air-dried and powdered whole plants of *H. serrata* were extracted with 60% EtOH for three times. The extract was partitioned between EtOAc and 1.0% HCl/H_2_O. Water-soluble materials, which were adjusted to pH 9 with 17% ammonia solution, were then extracted with CHCl_3_ to afford an alkaloidal extract. Further column chromatography (CC) over MCI gel, normal-phase silica gel, and Sephadex LH-20 led to the isolation of three new lycodine-type alkaloids (**1**–**3**), one new natural lycodine-type *Lycopodium* alkaloid (**4**), together with 11 known ones (**5**–**15**).

**Fig. 3:**
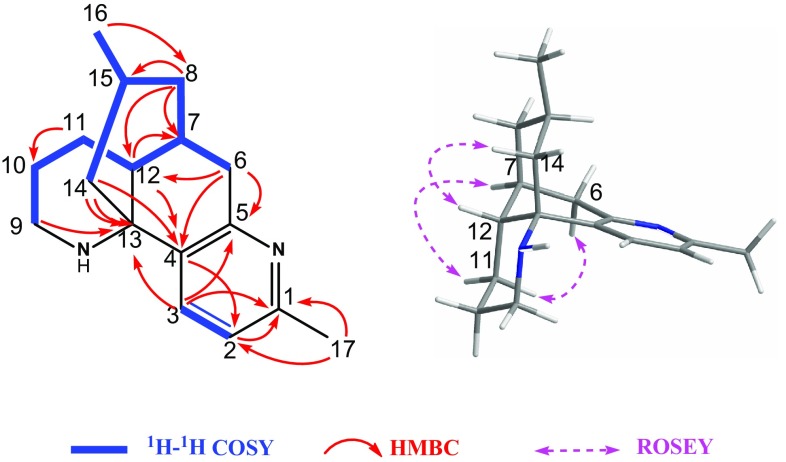
Key 2D NMR correlations of compound **1**

Compound **1** possessed a molecular formula of C_17_H_24_N_2_ as deduced from the HR–ESI–MS analysis ([M+H]^+^ *m/z* 257.2014, Calcd 257.2018), corresponding to seven degrees of unsaturation. Its IR spectrum showed strong absorption at 3433 and 1677 cm^−1^, indicated the NH and double bond groups, respectively. The existence of a pyridine moiety was revealed by the absorption bands at 209 and 273 nm in its UV spectrum and two characteristic proton signals [*δ*_H_ 8.31 and 7.07 (each 1H, d, *J* = 7.9 Hz)] in the downfield region of the ^1^H NMR spectrum. The ^13^C NMR and DEPT spectra exhibited 17 carbon resonances (Table 2), including three *sp* ^2^ quaternary carbons (*δ*_C_ 126.6, 157.7, and 157.8), one *sp* ^3^ quaternary carbon (*δ*_C_ 61.2), three *sp* ^3^ methines (*δ*_C_ 26.0, 33.1, and 41.6), two *sp* ^2^ methines (*δ*_C_ 122.0 and 133.5), six *sp* ^3^ methylenes (*δ*_C_ 23.3, 24.3, 35.0, 40.4, 42.7 and 47.4), and two methyls (*δ*_C_ 21.5 and 24.2) with corresponding protons as one double signal at *δ*_H_ 0.56 and one singlet signal at *δ*_H_ 2.55 (Table 1), respectively, in the ^1^H NMR spectrum. The above information suggested that **1** should be a lycodine-related *Lycopodium* alkaloid. Comparison of the ^1^H and ^13^C NMR spectroscopic data of **1** with those of the known alkaloid lycodine (**10**) [19] revealed that they were structural analogues. A major difference was the presence of one more methyl group [*δ*_H_ 2.55 (3H, s), *δ*_C_ 24.2] at C-1 in **1**, which was confirmed by the significant HMBC correlations from H-17 (*δ*_H_ 2.55) to C-1 (*δ*_C_ 157.8) and C-2 (*δ*_C_ 122.0) (Fig. 3), as well as the obvious triplet signal of H-1 in **10** was disappeared in **1**. It is worth noting that **1** was the first lycodine-type *Lycopodium* alkaloid possessing a methyl group at C-1.

**Table 1:** ^1^H NMR spectroscopic data of **1**–**5** (*δ* in ppm, *J* in Hz)

No.	**1** ^a^	**2** ^b^	**3** ^b^	**4** ^c^	**5** ^c^
2a	7.07 (d, 7.9)	2.37 (m)	6.39 (d, 9.5)	6.41 (d, 9.4)	6.43 (d, 9.4)
2b		2.31 (m)			
3a	8.31 (d, 7.9)	2.16 (2H, m)	7.94 (d, 9.5)	7.68 (d, 9.4)	7.90 (d, 9.4)
3b					
6a	3.17 (dd, 19.0, 7.2)	2.33 (dd, 17.8, 7.5)	2.89 (dd, 17.9, 5.4)	2.77 (dd, 17.0, 4.8)	4.60 (d, 5.2)
6b	2.76 (d, 19.0)	1.56 (d, 17.8)	2.30 (d, 17.9)	2.57 (d, 17.0)	
7	1.98 (overlapped)	1.94 (m)	2.48 (m)	3.63 (t, 4.8)	3.74 (dd, 5.2, 3.5)
8a	1.57 (overlapped)	3.93 (d, 2.6)	5.71 (br d, 4.3)	5.42 (d, 4.8)	5.55 (br d, 3.5)
8b	1.21 (overlapped)				
9a	3.54 (br d, 12.7)	2.76 (br d, 12.3)	2.61 (2H, overlapped)		
9b	2.84 (td, 12.7, 2.5)	2.42 (td, 12.3, 3.0)			
10a	1.97 (overlapped)	1.62 (2H, overlapped)	1.87 (dt, 12.6, 4.1)	1.71 (d, 6.8)	1.72 (d, 6.7)
10b	1.55 (overlapped)		1.27 (overlapped)		
11a	1.38 (br d, 16.7)	1.43 (2H, overlapped)	1.62 (m)	5.46 (q, 6.8)	5.63 (q, 6.7)
11b	1.11 (ddd, 16.7, 13.4, 3.9)		1.34 (ddd, 17.6, 12.6, 4.4)		
12	2.17 (d, 13.4)	2.14 (m)	2.09 (dt, 12.6, 4.1)		
14a	2.11 (br d, 11.9)	2.39 (d, 12.9)	2.65 (d, 16.7)	2.24 (d, 16.5)	2.29 (d, 16.8)
14b	1.87 (t, 11.9)	1.97 (d, 12.9)	1.94 (d, 16.7)	2.05 (d, 16.5)	2.16 (d, 16.8)
15	1.20 (overlapped)				
16a	0.56 (d, 7.8)	4.91 (br s)	3.79, 3.83 (ABq, 13.4)	1.53 (s)	1.58 (s)
16b		4.71 (br s)			
17	2.55 (s)				
N-CH_3_			2.69 (s)	2.09 (s)	

^a^Recorded at 500 MHz in C_5_D_5_N

^b^Recorded at 500 MHz in CD_3_OD

^c^Recorded at 600 MHz in CD_3_OD

The relative configuration of **1** was deduced by the ROESY experiment (Fig. 3) and the coupling constant. Biogenetially, H-12 and H-7 of the lycodine-type *Lycopodium* alkaloids were *β*- and *α*-orientated, respectively, which were also supported by the diagnostic ROESY correlations of H-6a with H-11b and H-7 with H-11a. Irradiation of H-12 enhanced the signal of H-14a, indicating that these protons were on the same facial plane. Furthermore, the methyl group at C-15 was located at an equatorial position by the large coupling constant (11.9 Hz) between H-14a and H-15. Thus, the structure of **1** was determined and named as 1-methyllycodine.

**Fig. 4:**
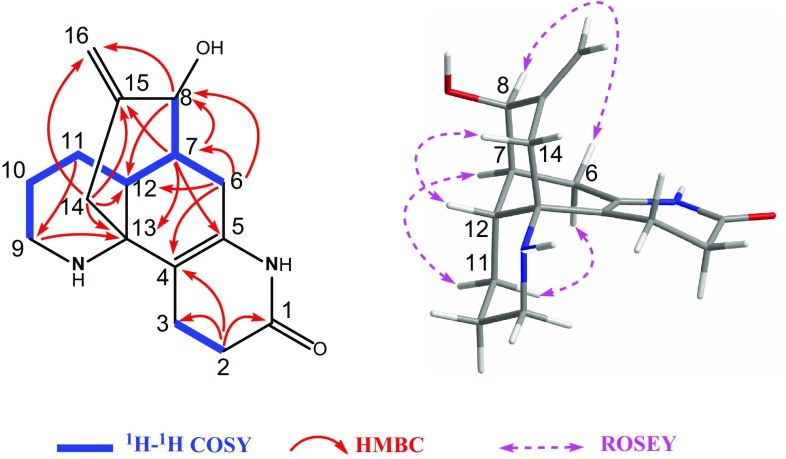
Key 2D NMR correlations of compound **2**

Compound **2** was assigned a molecular formula of C_16_H_22_N_2_O_2_ as determined by HR-EI-MS ([M]^+^ *m/z* 274.1674, Calcd 274.1681), requiring seven degrees of unsaturation. The IR spectrum was indicative of the presence of a hydroxy (3426 cm^−1^) and an amide carbonyl (1660 cm^−1^) groups. Its 1D NMR spectroscopic data (Tables 1, 2) showed general features analogous to those of des-*N*-methyl-*α*-obscurine [23, 27]. Differing from the latter, compound **2** has one more hydroxy group and a ∆^15^ double bond in its structure. The carbon resonance at *δ*_C_ 78.8 was assigned to C-8 bearing a hydroxy group, based on the HMBC correlations of H-8 (*δ*_H_ 3.93) with C-12 (*δ*_C_ 38.1), C-14 (*δ*_C_ 42.4), and C-16 (*δ*_C_ 115.1), and of H_2_-6 (*δ*_H_ 1.56 and 2.33) and H-7 (*δ*_H_ 1.94) with C-8, together with the ^1^H-^1^H COSY correlations of H-7/H-8 (Fig. 4). Moreover, the downfield shifts of C-15 (*δ*_C_ 147.4) and C-16 (*δ*_C_ 115.1) and the downfield shift of H-16 [*δ*_H_ 4.91 and 4.71 (each 1H, br s)] for **2** indicated the double bond was located between C-15 and C-16 at the terminal, which was also elucidated by the HMBC correlations of H-14b and H-8 with C-16, and of H-7 and H-14a (*δ*_H_ 2.39) with C-15.

**Table 2:** ^13^C NMR spectroscopic data of **1**–**5** (*δ* in ppm)

No.	**1** ^a^	**2** ^b^	**3** ^b^	**4** ^c^	**5** ^c^
1	157.8 s	173.6 s	165.8 s	165.6 s	165.3 s
2	122.0 d	31.6 t	118.3 d	118.1 d	119.1 d
3	133.5 d	19.8 t	143.1 d	142.3 d	141.3 d
4	126.6 s	111.6 s	121.9 s	120.7 s	123.7 s
5	157.7 s	131.3 s	144.1 s	145.9 s	145.9 s
6	35.0 t	28.0 t	30.0 t	35.2 t	72.1 d
7	33.1 d	41.6 d	35.3 d	34.3 d	42.2 d
8	42.7 t	78.8 d	126.3 d	125.8 d	121.3 d
9	40.4 t	43.3 t	51.8 t		
10	23.3 t	27.3 t	21.1 t	12.5 q	12.5 q
11	24.3 t	26.7 t	26.9 t	114.3 d	115.0 d
12	41.6 d	38.1 d	34.3 d	137.3 s	140.3 s
13	61.2 s	58.2 s	58.3 s	61.1 s	55.7 s
14	47.4 t	42.4 t	39.9 t	50.7 t	50.2 t
15	26.0 d	147.4 s	137.8 s	135.4 s	136.6 s
16	21.5 q	115.1 t	66.4 t	22.7 q	22.9 q
17	24.2 q				
N-CH_3_			38.0 q	29.7 q	

^a^Recorded at 125 MHz in C_5_D_5_N

^b^Recorded at 125 MHz in CD_3_OD

^c^Recorded at 150 MHz in CD_3_OD

In the ROESY spectrum (Fig. 4), the *β* **-** and *α*-orientation of H-12 and H-7, respectively, were revealed by the observed correlations of H-6a/H-11b, H-7/H-11a, and H-12/H-14b. Furthermore, the obvious ROESY correlation of H-8/H-6b allowed the assignment of OH-8 as *α* **-**orientated. On the basis of the above evidence, the structure of **2** was assigned as 8*α*-hydroxy-15,16-dehydro-des-*N*-methyl-*α*-obscurine.

Compound **3** showed the pseudo-molecular ion peak at *m/z* 287.1759 [M+H]^+^ (Calcd 287.1760) in the HR–ESI–MS, which established a molecular formula of C_17_H_22_N_2_O_2_, indicating eight degrees of unsaturation. IR absorptions implied the presence of amide carbonyl (1657 cm^−1^) and hydroxy (3421 cm^−1^) functionalities. The absorption bands at 231 and 310 nm in its UV spectrum and two characteristic proton signals [*δ*_H_ 7.94 and 6.39 (each 1H, d, *J* = 9.5 Hz)] in the low field region of the ^1^H NMR spectrum revealed the existence of an *α*-pyridone moiety. Additionally, an *N*-methyl group [*δ*_H_ 2.69 (3H, s), *δ*_C_ 38.0] was also displayed in the ^1^H, ^13^C NMR, and DEPT spectra (Tables 1, 2). Analysis of the 1D NMR spectra of **3** revealed that its spectroscopic data closely resembled those of 16-hydroxyhuperzine B (**15**) [25], a known lycodine-type alkaloid previously isolated from *Lycopodium casuarinoides*. The only difference between those two compounds was that **3** possessed an additional *N*-methyl group. Consequently, compound **3** was assumed to be the *N*-methylated derivative of 16-hydroxyhuperzine B, which was confirmed by observed key HMBC correlations from *N*-methyl (H-17) at *δ*_H_ 2.69 to C-9 (*δ*_C_ 51.8) and C-13 (*δ*_C_ 58.3). The observed ROESY correlations of H-6a/H-11b, H-7/H-11a, and H-12/H-14b indicated that H-12 and H-7 were *β* **-** and *α*-orientated, respectively. Hence, the structure of **3** was elucidated as *N*-methyl-16-hydroxyhuperzine B.

**Fig. 5:**
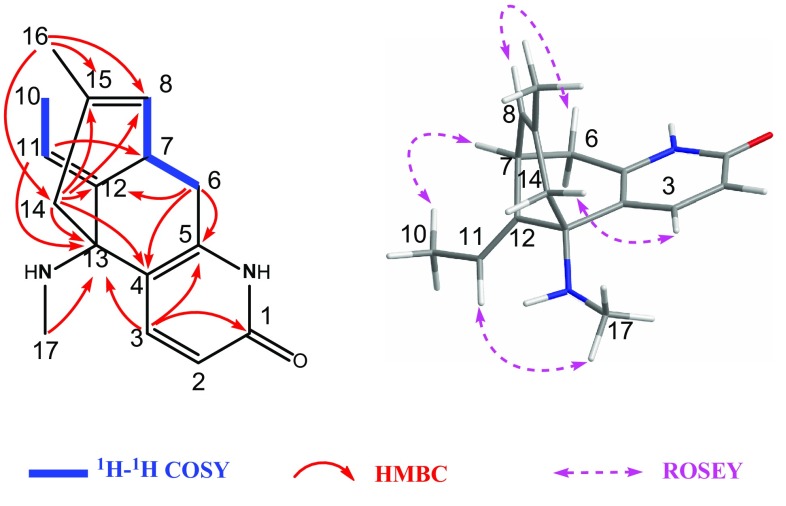
Key 2D NMR correlations of compound **4**

Compound **4** was assigned with a molecular ion peak at *m/z* 257.1653 [M+H]^+^ (Calcd 257.1654) in the HR–ESI–MS, coincided with the molecular formula of C_16_H_20_N_2_O, which required eight degrees of unsaturation. IR spectrum revealed the presence of NH (3431 cm^−1^) and amide carbonyl (1657 cm^−1^) groups. The ^13^C NMR and DEPT spectra displayed 16 carbon signals (Table 2), including five *sp* ^2^ quaternary carbons [one carbonyl (*δ*_C_ 165.6) and four olefinic (*δ*_C_ 120.7, 135.4, 137.3, and 145.9)], one *sp* ^3^ quaternary carbon (*δ*_C_ 61.1), one *sp* ^3^ methines (*δ*_C_ 34.3), four *sp* ^2^ methines (*δ*_C_ 114.3, 118.1, 125.8, and 142.3), two *sp* ^3^ methylenes (*δ*_C_ 35.2 and 50.7), and three methyls (*δ*_C_ 12.5, 22.7, and 29.7) with corresponding protons as double signal at *δ*_H_ 1.71 and singlet signals at *δ*_H_ 1.53 and 2.09, respectively, in the ^1^H NMR spectrum (Table 1). The above data allowed **4** to be a *N*-methyl derivative of huperzine A, which was confirmed by the observed key HMBC correlation between the proton at *δ*_H_ 2.09 with the *sp* ^*3*^ quaternary carbon C-13 at *δ*_C_ 61.1 (Fig. 5). The observed ROESY correlations (Fig. 5) of H-6b/H-8 and of H-7/H-10 (*δ*_H_ 1.71) indicated that H-7 was *α*-orientated. The *R* ^*^ configuration of C-13 was assigned by the clear ROESY correlations of H-3 with H-14a and H-11 with H-17. Accordingly, the structure of **4** was characterized as *N*-methylhuperzine A, which reported previously as a synthetic product from the methylation of huperzine A [2, 9]. To our knowledge, compound **4** was isolated for the first time from natural resources.

Compounds **1**-**5** were evaluated for their *β*-site amyloid precursor protein (APP) cleaving enzyme 1 (BACE1) inhibitory activity. Unfortunately, the results showed that all the compounds were inactive (IC_50_ values >100 *μ*M).

## Experimental Section

### Gernal Experimental Procedures

UV spectra were recorded with a Shimadzu UV-2401A spectrophotometer. IR spectra were recorded on Bruker Tensor 27 spectrometer with KBr pellets. 1D and 2D NMR spectra were carried out on Bruker AM-400, DRX-500, or AVANCE III-600 spectrometers. Chemical shifts were reported using TMS as the internal standard. ESI–MS were run on Shimadzu UPLC–IT–TOF–MS instrument. HR–ESI–MS spectra were measured using Agilent G 6230 TOF MS (Agilent). EI–MS and HR–EI–MS spectra were measured with a Waters AutoSpec Premier P776 mass spectrometer (Waters, Milford, MA, USA).Crystal analysis were performed on a Bruker APEX DUO diffractometer equipped with an APEX II CCD, using Cu K*α* radiation (*λ* = 1.54178 Å). Cell refinement and data reduction were performed with Bruker SAINT. Column chromatography (CC) was performed on silica gel (100–200 or 200–300 mesh; Qingdao Marine Chemical Co. Ltd., Qingdao, China), Sephadex LH-20 (GE Healthcare Bio-Sciences AB, Sala, Sweden) and MPLC was performed on a Lisui EZ Purify III System packed with MCI gel (CHP20P, 75–150 mm; Mitsubishi Chemical Corporation, Tokyo, Japan). Precoated silica gel GF254 plates (Qingdao Haiyang Chemical Co. Ltd.) were used for thin-layer chromatography (TLC). Fractions were monitored by TLC and spots were visualized by Dragendorff’s reagent.

### Plant Material

The club moss *H. serrata* was collected from Taijiang County, Guizhou Province, People’s Republic of China in July, 2012. The plant was identified by one of the authors, Prof. Lu-Tai Pan (Guiyang College of Traditional Chinese Medicine). And the voucher specimen (No. 20120312h) was deposited at the State Key Laboratory of Phytochemistry and Plant Resources in West China, Kunming Institute of Botany, Chinese Academy of Sciences.

### Extraction and Isolation

The aerial parts of club moss *H. serrata* (40 kg) were chopped into sections and extracted with 60% EtOH/H_2_O under reflux for three times (24 h × 3). The resultant extract was partitioned between EtOAc and 1% HCl/H_2_O solution to afford ethyl acetate and water soluble fractions, respectively. The water-soluble fractions were adjusted to pH 9 with 17% ammonia solution, and then extracted with CHCl_3_ to give an alkaloidal extract (106 g). The alkaloidal extract was subjected to a MCI gel CC (MeOH/H_2_O, 5 to 100%) to afford fractions I–V. Fraction I (18.0 g) was further chromatographed over a silica gel CC (CHCl_3_/MeOH, 50:1 → 5:1) to give subfractions (Fr. I–I to Fr. I–III). Fr. I–II (3.4 g) was subjected to a silica gel CC (EtOAc/MeOH, 25:1 → 5:1) to afford compounds **8** (5.3 mg) and **9** (35.0 mg). From Fr. I–III (18.6 g), compounds **7** (12.5 mg) and **10** (10.8 mg) were obtained after purified by a silica gel CC (CHCl_3_/MeOH, 30:1 → 10:1). Fraction II (15.0 g) was subjected to a silica gel CC eluted with petroleum ether (PE)/EtOAc/Et_2_NH, 100:2:1 → 50:50:1, to give subfractions (Fr. II–I to Fr. II–III). Fr. II–I (2.4 g) was separated over a silica gel CC (EtOAc/MeOH, 15:1 → 5:1) to yield compounds **2** (9.5 mg), **4** (5.3 mg), and **6** (18.7 mg). Fr. II–II (1.6 g) was purified by CC over a silica gel (CHCl_3_/MeOH, 40:1 → 1:1) to give compound **3** (30.1 mg). Fraction III (12.9 g) was subjected to a Sephadex LH-20 CC (MeOH) to yield subfractions (Fr. III–I to Fr. III–III). Fr. III–II (3.5 g) was chromatographed on a silica gel CC (CHCl_3_/EtOAc/MeOH, 20:5:1 → 5:5:1) to afford compounds **12** (16.0 mg) and **13** (3.7 mg). Fraction IV (11.4 g) was performed on repeated silica gel CC (PE/acetone/Et_2_NH, 80:1:1 → 50:5:1 and then EtOAc/MeOH, 35:1 → 10:1) to provide subfractions (Fr. IV–I to Fr. IV–III). Fraction IV–I (2.5 g) was chromatographed over a silica gel CC (CHCl_3_/MeOH, 20:1 → 5:1) to furnish compound **14** (16.4 mg). Fraction IV–II (3.9 g) was submitted to a Sephadex LH-20 CC (MeOH) and further purified via a silica gel CC (PE/acetone/Et_2_NH, 50:1:1 → 50:10:1) to produce compounds **1** (8.7 mg) and **15** (8.7 mg). The last Fraction V (9.3 g) was applied to repeated silica gel CC (CHCl_3_/acetone, 30:1 → 1:1 and then PE/EtOAc, 10:1 → 1:1) and purified via a Sephadex LH-20 CC (MeOH) to yield compounds **5** (18.5 mg) and **11** (6.6 mg).

#### 1-Methyllycodine (**1**)

Colorless oil; $\left[ {\upalpha} \right]_{{\text{D}}}^{{21.4}}$ – 8.8 (*c* = 0.12, MeOH); UV (MeOH) *λ*_max_ (log *ε*) 209 (3.56), 273 (3.40), 378 (1.91), 452 (1.56), 570 (0.91) nm; IR (KBr) *ν*_max_ 3433, 2929, 1677, 1429, 1204, 1134, 837, and 722 cm^−1^; ^1^H NMR and ^13^C NMR data see Tables 1 and 2; ESIMS (positive) *m/z* 257 [M+H]^+^; HRESIMS (positive) *m/z* 257.2014 [M+H]^+^ (calcd for C_17_H_24_N_2_, 257.2018).

#### 8α-Hydroxy-15,16-dehydro-des-N-methyl-α-obscurine (**2**)

Colorless solid; mp 264–265 °C; $\left[ {\upalpha} \right]_{{\text{D}}}^{{22.3}}$ – 40.4 (*c* = 0.11, MeOH); UV (MeOH) *λ*_max_ (log *ε*) 202 (3.64), 254 (3.73) nm; IR (KBr) *ν*_max_ 3426, 2926, 1660, 1440, 1383, 1220, 1026, 905, and 651 cm^−1^; ^1^H NMR and ^13^C NMR data see Tables 1 and 2; EIMS *m/z* 274 [M]^+^ (10), 260 (8), 203 (100), 175 (41), 91 (10); HREIMS (positive) *m/z* 274.1674 [M]^+^ (calcd for C_16_H_22_N_2_O_2_, 274.1681).

#### N-Methyl-16-hydroxyhuperzine B (**3**)

Colorless solid; mp 200–201 °C; $\left[ {\upalpha} \right]_{{\text{D}}}^{{21.3}}$ – 44.8 (*c* = 0.12, MeOH); UV (MeOH) *λ*_max_ (log *ε*) 203 (4.00), 231 (3.95), 310 (3.82) nm; IR (KBr) *ν*_max_ 3421, 2929, 1657, 1453, 1304, 1105, 837, and 515 cm^−1^; ^1^H NMR and ^13^C NMR data see Tables 1 and 2; ESIMS (positive) *m/z* 287 [M+H]^+^; HRESIMS (positive) *m/z* 287.1759 [M+H]^+^ (calcd for C_17_H_22_N_2_O_2_, 287.1760).

#### N-Methylhuperzine A (**4**)

Colorless solid; mp 235–236 °C; $\left[ {\upalpha} \right]_{{\text{D}}}^{{22.9}}$ – 97.6 (*c* = 0.11, MeOH); UV (MeOH) *λ*_max_ (log *ε*) 202 (3.95), 232 (3.97), 312 (3.86) nm; IR (KBr) *ν*_max_ 3431, 2927, 1657, 1610, 1441, 1121, 839, and 652 cm^−1^; ^1^H NMR and ^13^C NMR data see Tables 1 and 2; ESIMS (positive) *m/z* 257 [M+H]^+^; HRESIMS (positive) *m/z* 257.1653 [M+H]^+^ (calcd for C_16_H_20_N_2_O, 257.1654).

#### 6β-Hydroxyhuperzine A (**5**)

Colorless crystals; mp 207–210 °C; $\left[ {\upalpha} \right]_{{\text{D}}}^{{23.5}}$ – 145.2 (*c* = 0.11, MeOH); UV (MeOH) *λ*_max_ (log *ε*) 203 (3.93), 232 (3.80), 310 (3.65) nm; IR (KBr) *ν*_max_ 3415, 2926, 1655, 1598, 1441, 1383, 1090, 841, and 621 cm^−1^; ^1^H NMR and ^13^C NMR data see Tables 1 and 2; ESIMS (positive) *m/z* 259 [M+H]^+^; HRESIMS (positive) *m/z* 259.1443 [M+H]^+^ (calcd for C_15_H_18_N_2_O_2_, 259.1447).

### X-ray Crystal Structure Analysis

Crystal analysis were performed on a Bruker APEX DUO diffractometer equipped with an APEX II CCD, using Cu K*α* radiation (*λ* = 1.54178 Å). Cell refinement and data reduction were performed with Bruker SAINT.

The structure of **5** was solved by direct methods using SHELXS-97. Refinements were performed with SHELXL-97 using full-matrix least-squares, with anisotropic displacement parameters for all the non-hydrogen atoms. The H-atoms were placed in calculated positions and refined using a riding model. Crystallographic data for **5** in this paper have been deposited with the Cambridge Crystallographic Data Centre (CCDC 1518517). Copies of the data can be obtained free of charge from the CCDC via www.ccdc.cam.ac.uk.

#### X-ray Crystal Data for 6β-Hydroxyhuperzine A (**5**)

C_15_H_18_N_2_O_2_·H_2_O, *M* = 276.33, *a* = 14.227(7) Å, *b* = 12.474(6) Å, *c* = 8.948(5) Å, *α* = 90°, *β* = 113.406(7)°, *γ* = 90°, *V* = 1457.4(13) Å^3^, *T* = 100(2) K, space group *C*2, *Z* = 4, *μ*(MoKα) = 0.088 mm^−1^, 6582 reflections measured, 3687 independent reflections (*R*_*int*_ = 0.0400). The final *R*_*1*_ values were 0.0840 (*I* > 2*σ*(*I*)). The final *wR*(*F* ^2^) values were 0.2254 (*I* > 2*σ*(*I*)). The final *R*_*1*_ values were 0.0914 (all data). The final *wR*(*F* ^2^) values were 0.2326 (all data). The goodness of fit on *F* ^2^ was 1.168. Flack parameter = 0.3(7).

### BACE1 Inhibitory Activity Assay

Compounds **1**–**5** were assessed for *β*-site amyloid precursor protein (APP) cleaving enzyme 1 (BACE1) inhibitory activity. BACE1 inhibitory evaluation was tested using a fluorescence resonance energy transfer (FRET) assay kit supplied by PanVera (Kit P2985, Madison, WI, USA). The kit was using purified baculovirus expression BACE1 and substrates of a new red FRET peptide substrates, which were based on the “Swedish” mutation. The BACE1 FRET assay was carried out according to the principle described in Ref. [26]. The first orally available non-peptidic *β*-secretase inhibitor LY2811376, [28] which had an IC_50_ value of 401.21 nM, was using as a positive control.

## Electronic supplementary material

Below is the link to the electronic supplementary material.

Supplementary material 1 (DOCX 1683 kb)
