# Supplementary material for: Lycodine-Type Lycopodium Alkaloids from the Whole Plants of Huperzia serrata
Source: Nat Prod Bioprospect. 2017 Jul 25;7(5):405–11. doi: 10.1007/s13659-017-0140-z (PMC5655362; doi:10.1007/s13659-017-0140-z)
Supplement: Supplementary file 1 — Supplementary material 1 (DOCX 1683 kb) [file 13659_2017_140_MOESM1_ESM.docx]

**Lycodine-type *Lycopodium* Alkaloids from The Whole Plants of *Huperzia serrata***

Yu-Chen Liu ∙ Zhi-Jun Zhang ∙ Jia Su ∙ Li-Yan Peng ∙ Lu-Tai Pan ∙ Xing-De Wu ∙ Qin-Shi Zhao

Y.-C Liu ∙ Z.-J. Zhang ∙ J. Su ∙ L.-Y. Peng ∙ X.-D. Wu (🖂)∙ Q.-S. Zhao (🖂)

State Key Laboratory of Phytochemistry and Plant Resources in West China, Kunming Institute of Botany, Chinese Academy of Sciences, Kunming 650201, People’s Republic of China.

email: qinshizhao@mail.kib.ac.cn, [wuxingde@mail.kib.ac.cn](mailto:wuxingde@mail.kib.ac.cn)

L.-T. Pan

Guiyang College of Traditional Chinese Medicine

Y.-C. Liu ∙ Z.-J. Zhang

University of Chinese Academy of Sciences, Beijing 100039, People’s Republic of China

**Table of Contents**

| **Page** | **Contents** |
| --- | --- |
| 3-9 | Figure S1-S7. ^1^H NMR, ^13^C NMR, DEPT, HSQC, HMBC, ^1^H-^1^H COSY, ROESY, and HR-ESI-MS spectra of the compound **1** |
| 10-16 | Figure S8-S14. ^1^H NMR, ^13^C NMR, DEPT, HSQC, HMBC, ^1^H-^1^H COSY, ROESY, and HR-EI-MS spectra of the compound **2** |
| 17-23 | Figure S15-S21. ^1^H NMR, ^13^C NMR, DEPT, HSQC, HMBC, ^1^H-^1^H COSY, ROESY, and HR-ESI-MS spectra of the compound **3** |
| 24-30 | Figure S22-S28. ^1^H NMR, ^13^C NMR, DEPT, HSQC, HMBC, ^1^H-^1^H COSY, ROESY, and HR-ESI-MS spectra of the compound **4** |
| 31-37 | Figure S29-S35. ^1^H NMR, ^13^C NMR, DEPT, HSQC, HMBC, ^1^H-^1^H COSY, ROESY, and HR-ESI-MS spectra of the compound **5** |
| 38-40 | The single-crystal X-ray diffraction of the compound **5** |


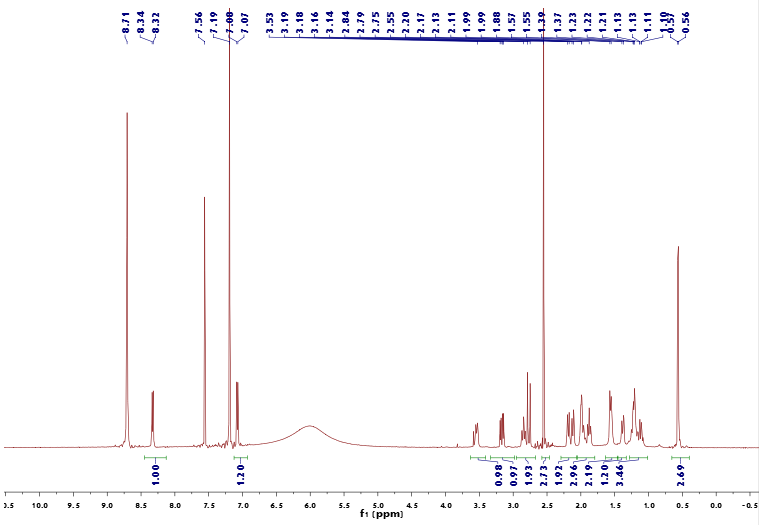


Figure S1. ^1^H NMR (500 MHz, C_5_D_5_N) spectrum of the compound **1**


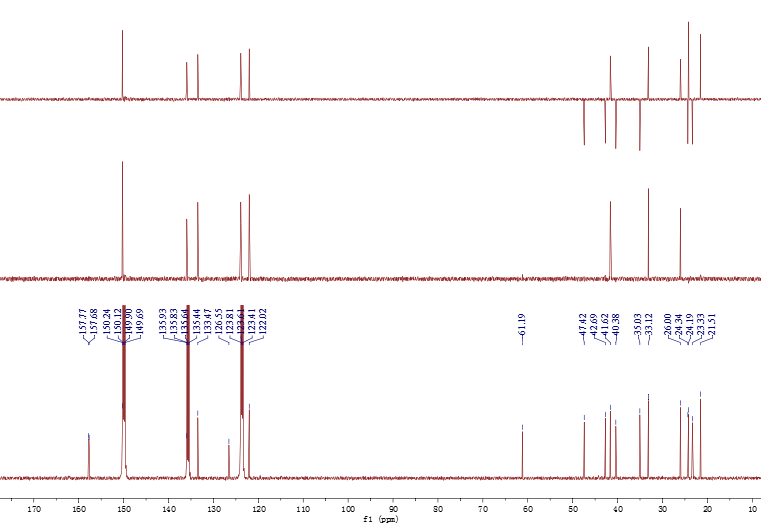


Figure S2. ^13^C NMR and DEPT (125 MHz, C_5_D_5_N) spectrum of the compound **1**


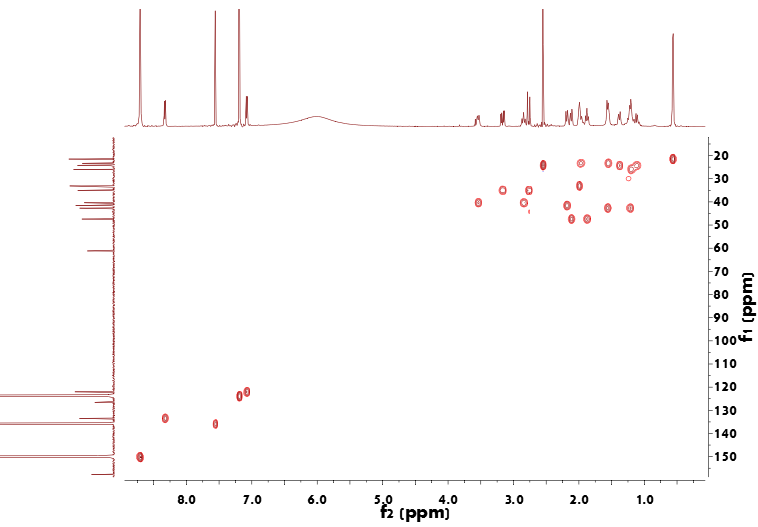


Figure S3. HSQC spectrum of the compound **1**


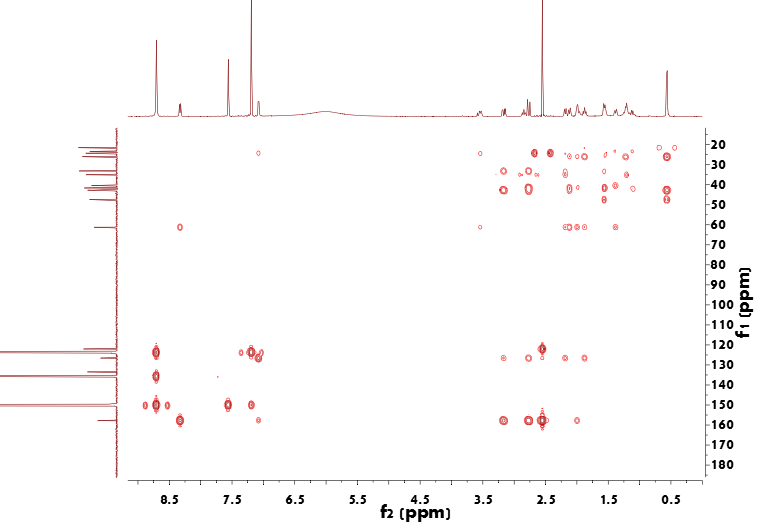


Figure S4. HMBC spectrum of the compound **1**


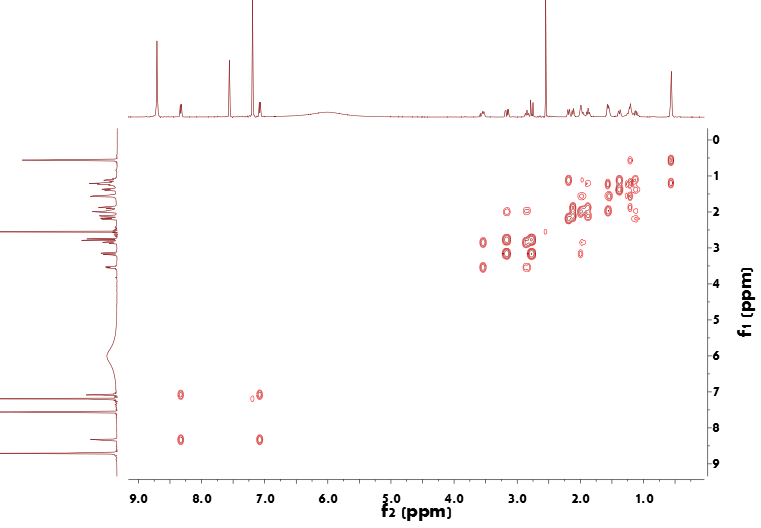


Figure S5. ^1^H-^1^H COSY spectrum of the compound **1**


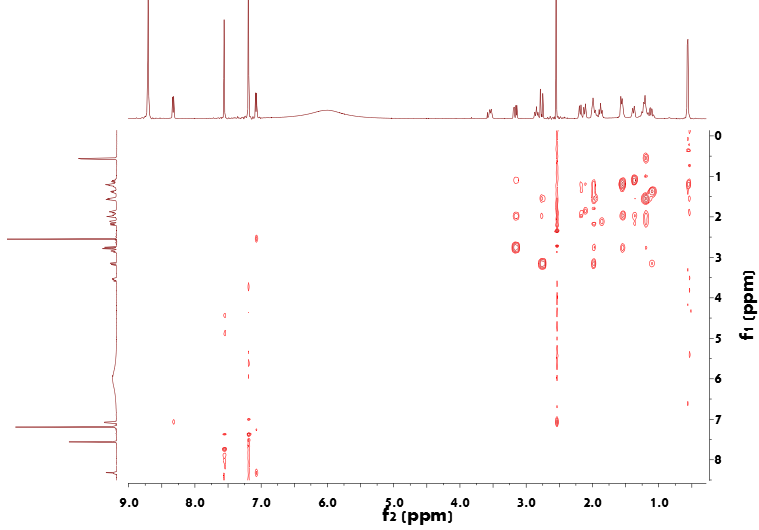


Figure S6. ROESY spectrum of the compound **1**


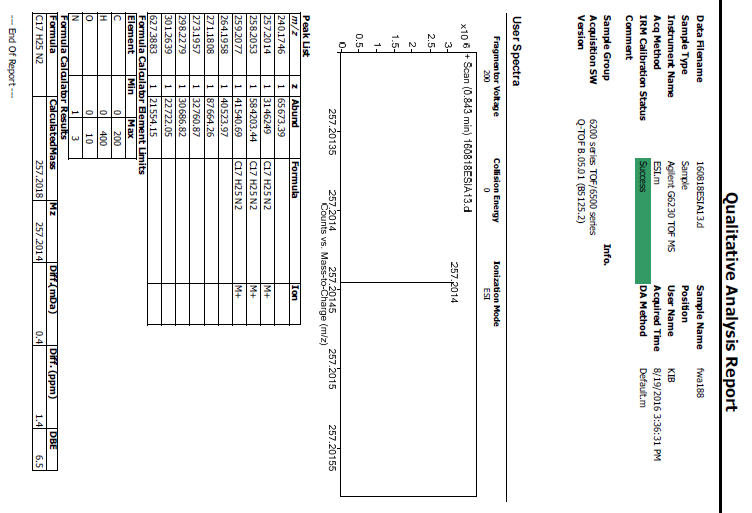


Figure S7. HR-ESI-MS spectrum of the compound **1**


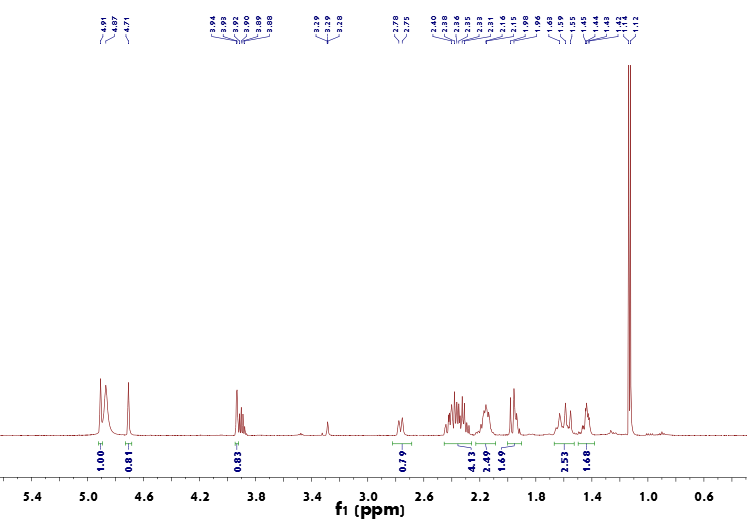


Figure S8. ^1^H NMR (500 MHz, CD_3_OD) spectrum of the compound **2**


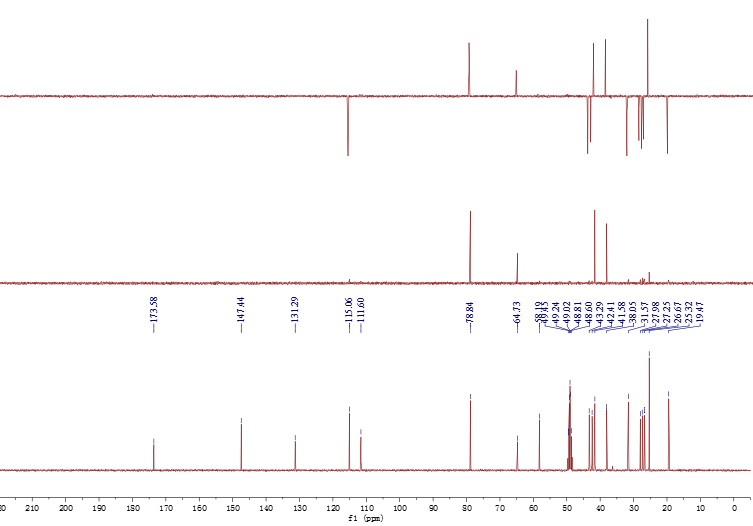


Figure S9. ^13^C NMR and DEPT (125 MHz, CD_3_OD) spectrum of the compound **2**


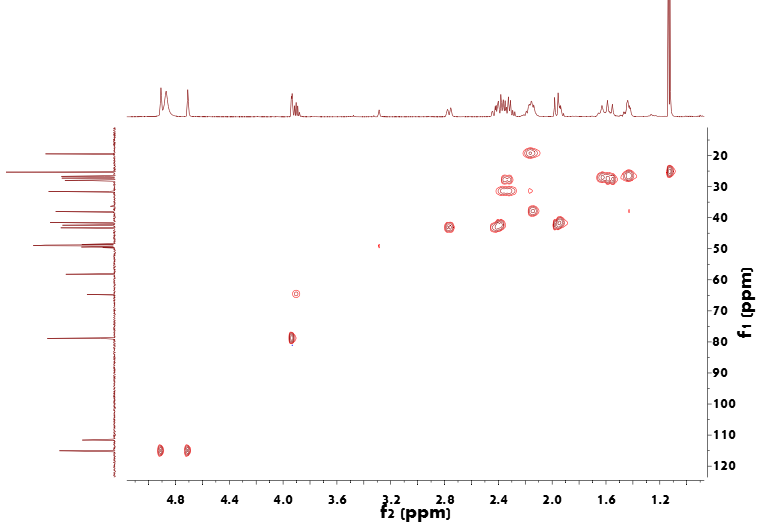


Figure S10. HSQC spectrum of the compound **2**


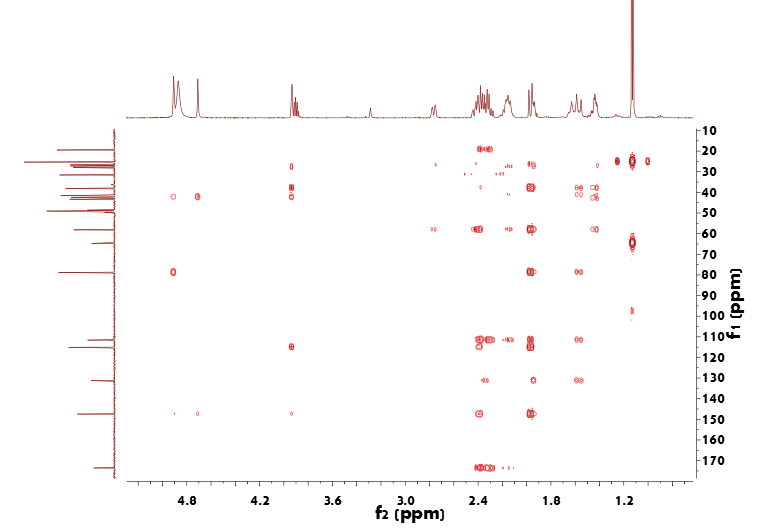


Figure S11. HMBC spectrum of the compound **2**


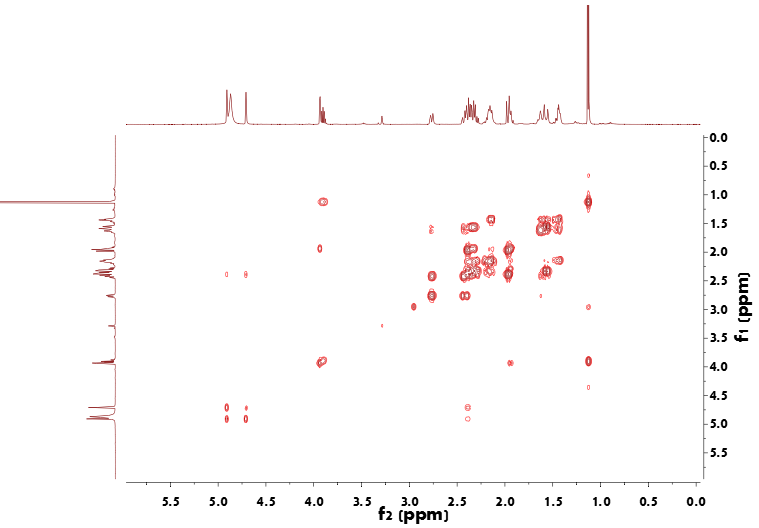


Figure S12. ^1^H-^1^H COSY spectrum of the compound **2**


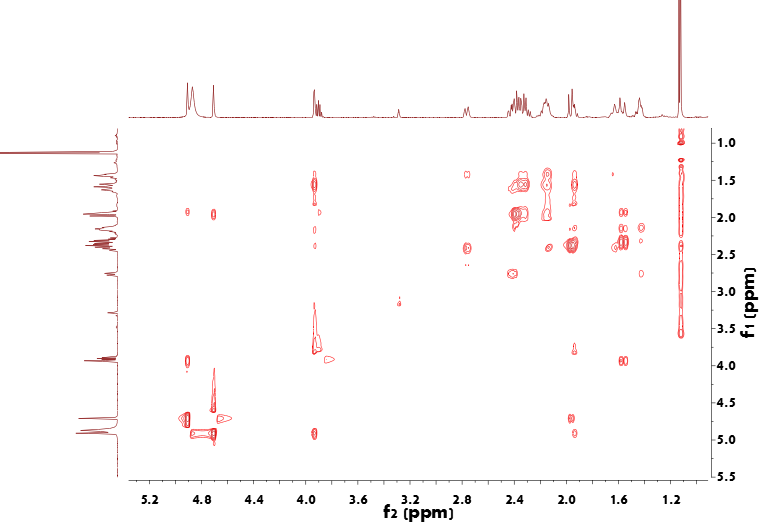


Figure S13. ROESY spectrum of the compound **2**


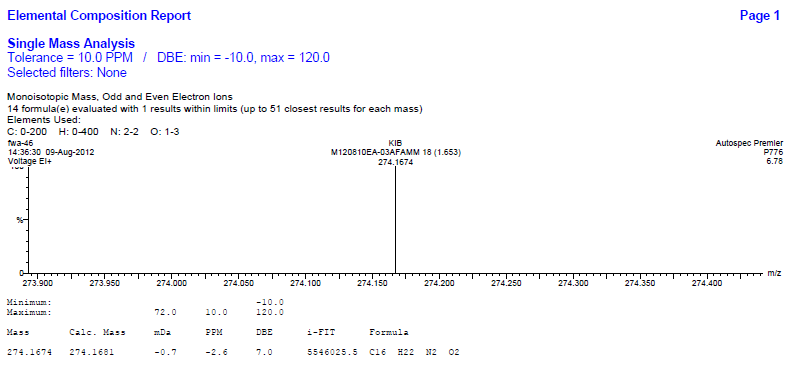


Figure S14. HR-EI-MS spectrum of the compound **2**


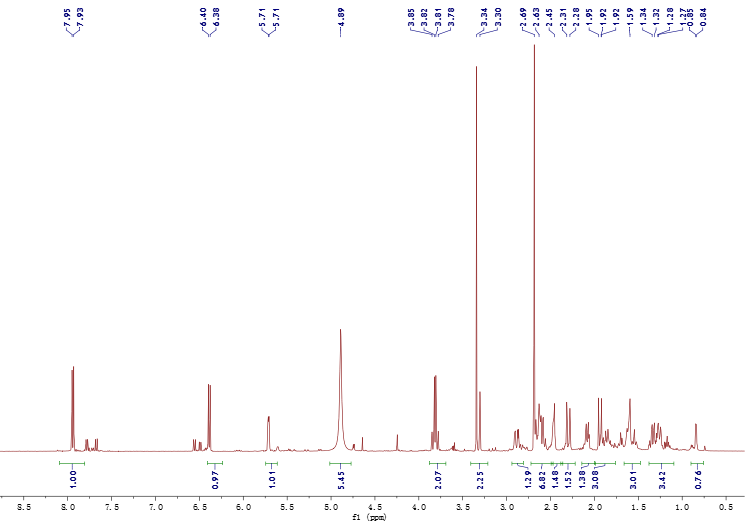


Figure S15. ^1^H NMR (500 MHz, CD_3_OD) spectrum of the compound **3**


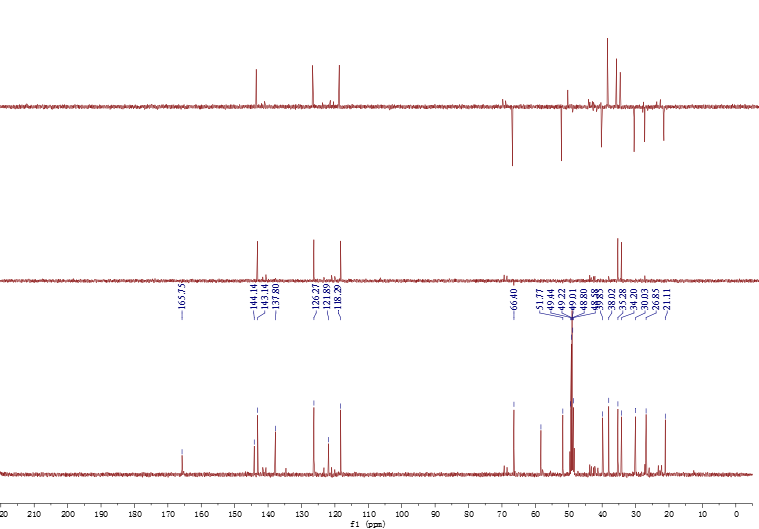


Figure S16. ^13^C NMR and DEPT (125 MHz, CD_3_OD) spectrum of the compound **3**


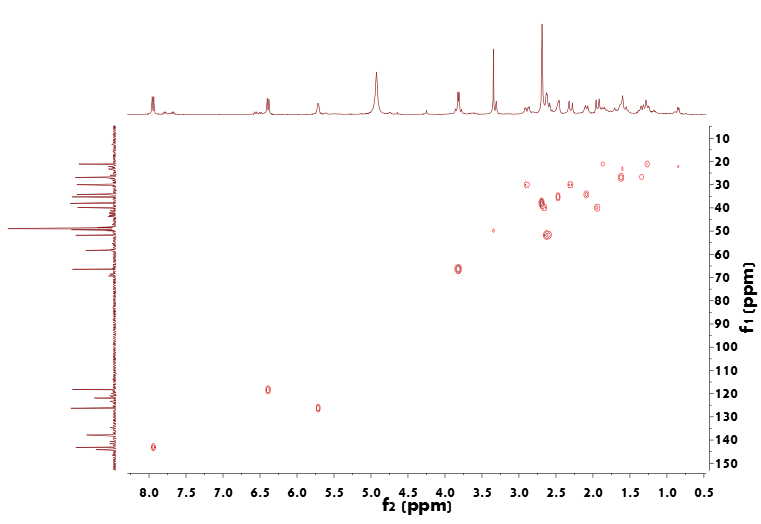


Figure S17. HSQC spectrum of the compound **3**


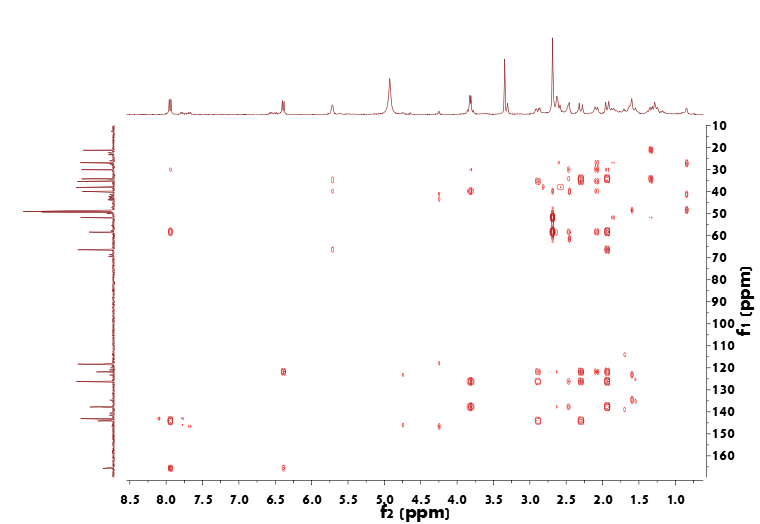


Figure S18. HMBC spectrum of the compound **3**


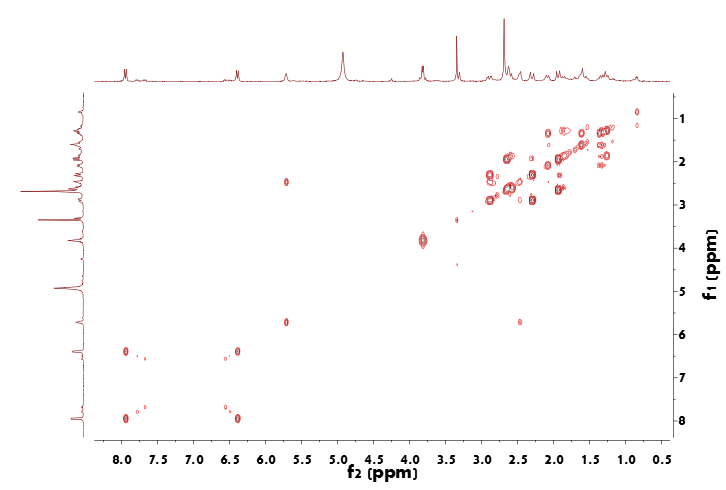


Figure S19. ^1^H-^1^H COSY spectrum of the compound **3**


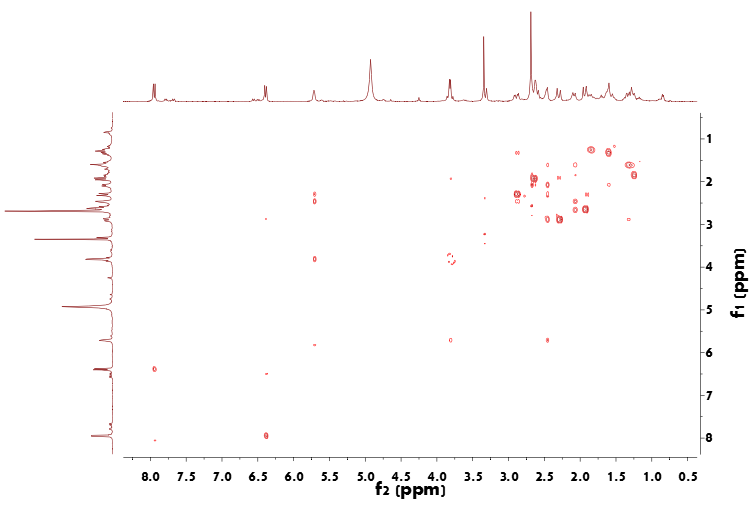


Figure S20. ROESY spectrum of the compound **3**


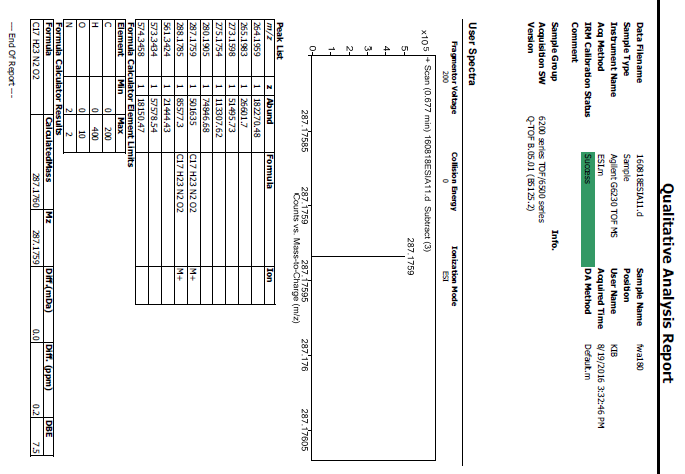


Figure S21. HR-ESI-MS spectrum of the compound **3**


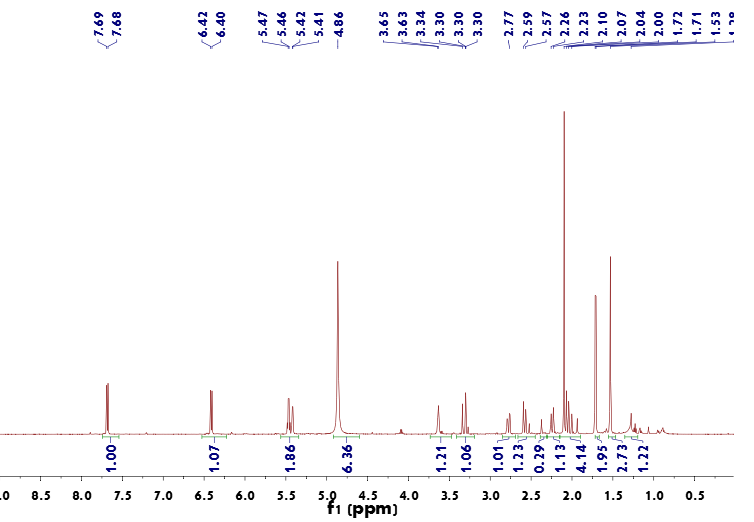


Figure S22. ^1^H NMR (600 MHz, CD_3_OD) spectrum of the compound **4**


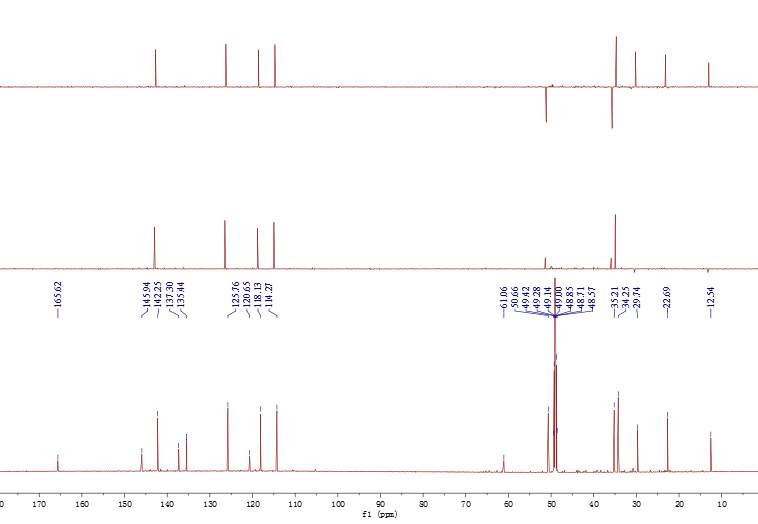


Figure S23. ^13^C NMR and DEPT (150 MHz, CD_3_OD) spectrum of the compound **4**


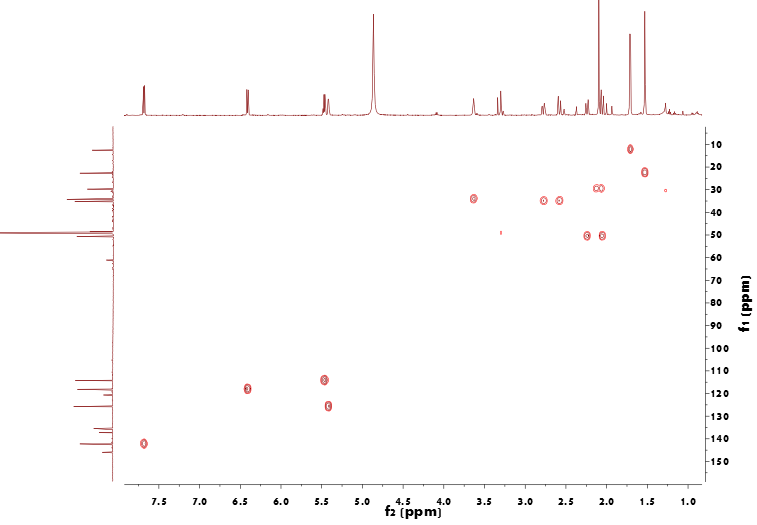


Figure S24. HSQC spectrum of the compound **4**


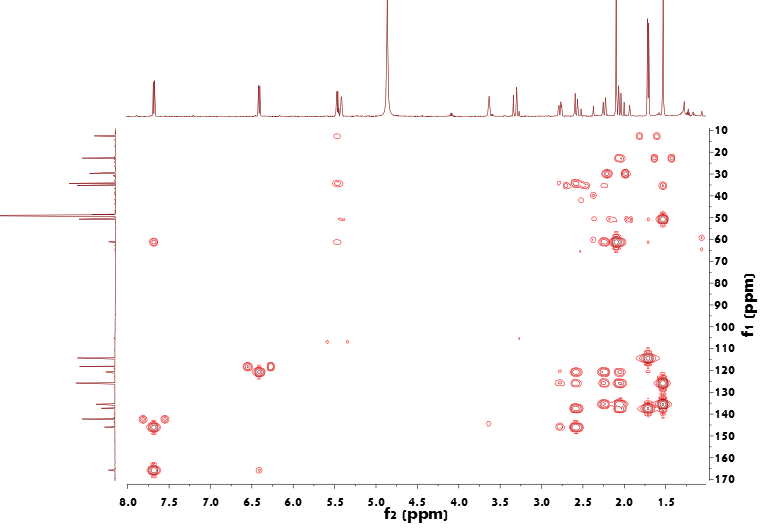


Figure S25. HMBC spectrum of the compound **4**


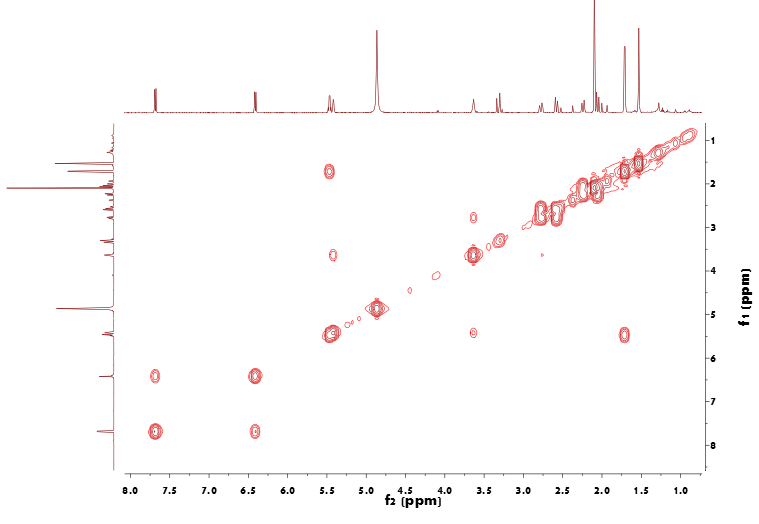


Figure S26. ^1^H-^1^H COSY spectrum of the compound **4**


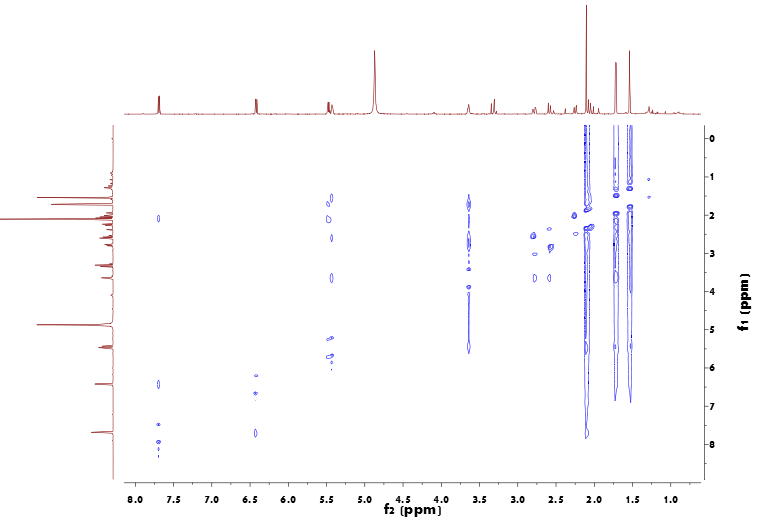


Figure S27. ROESY spectrum of the compound **4**


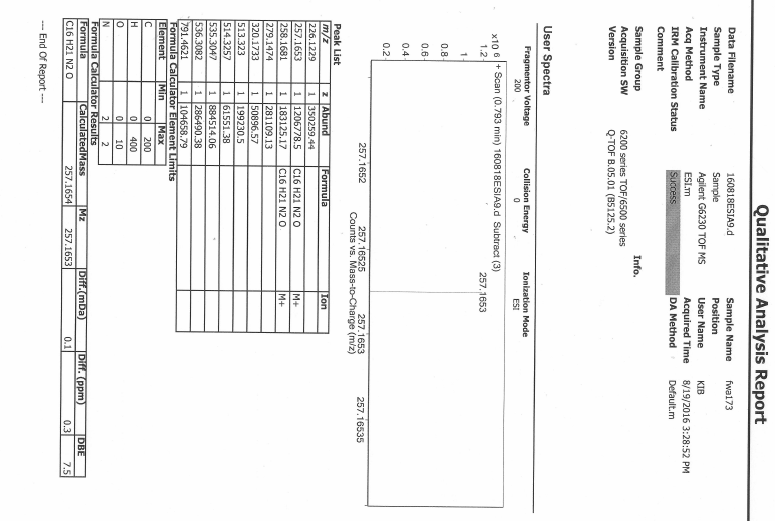


Figure S28. HR-ESI-MS spectrum of the compound **4**


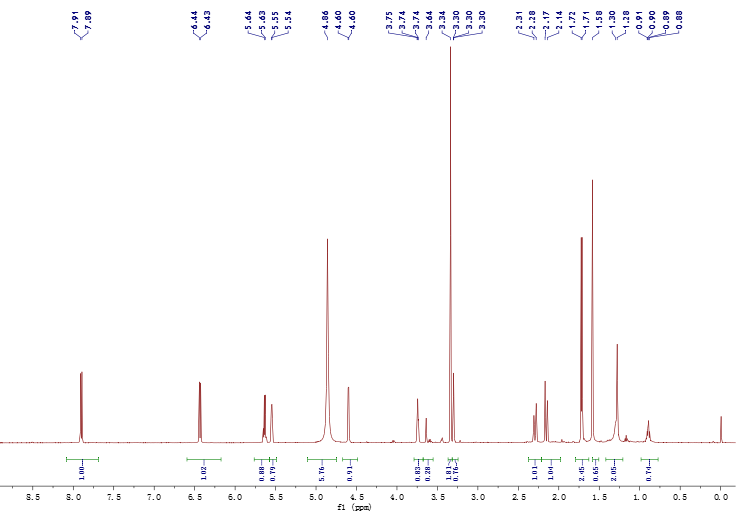


Figure S29. ^1^H NMR (600 MHz, CD_3_OD) spectrum of the compound **5**


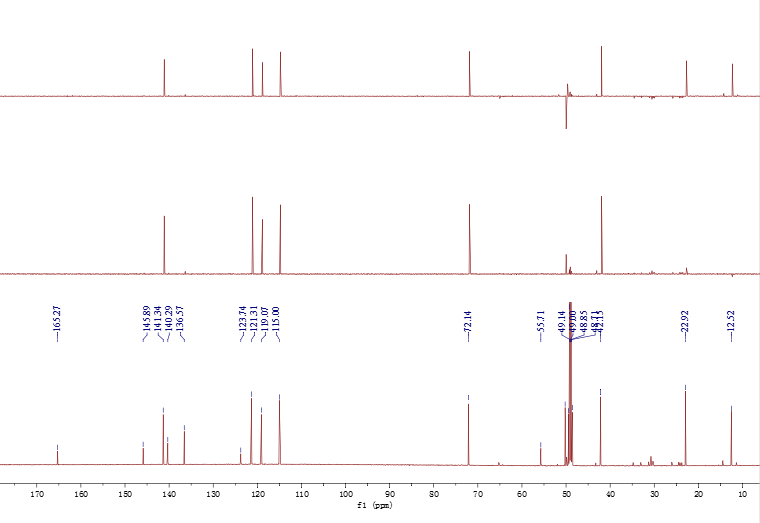


Figure S30. ^13^C NMR and DEPT (150 MHz, CD_3_OD) spectrum of the compound **5**


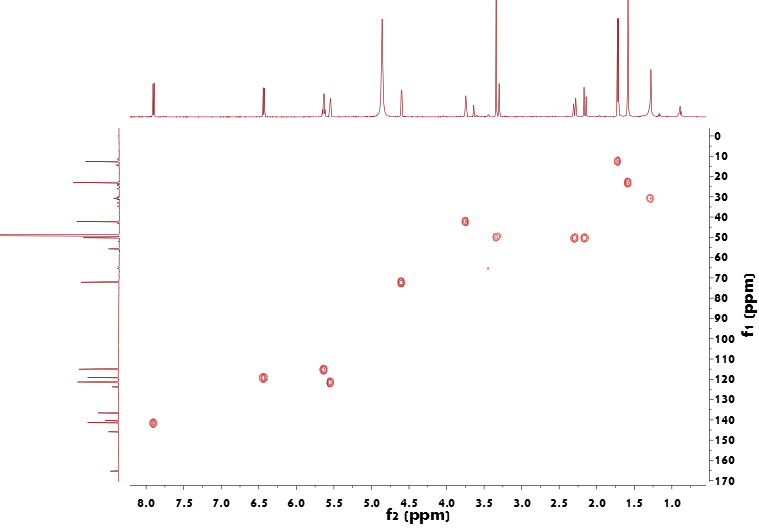


Figure S31. HSQC spectrum of the compound **5**


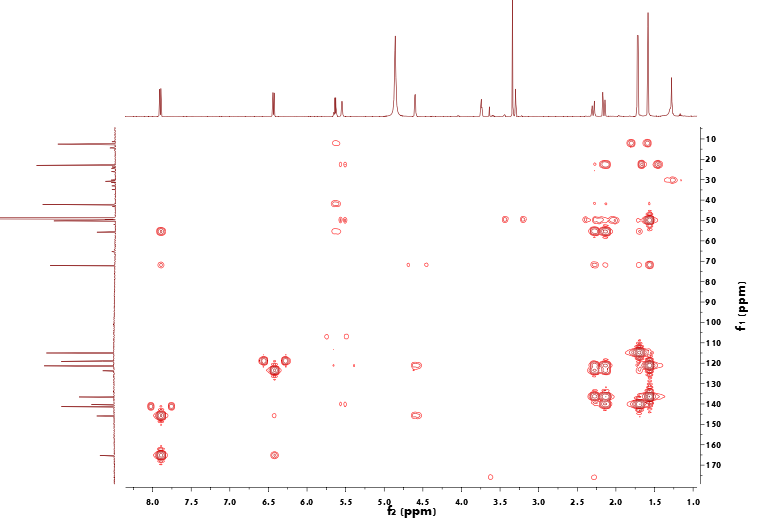


Figure S32. HMBC spectrum of the compound **5**


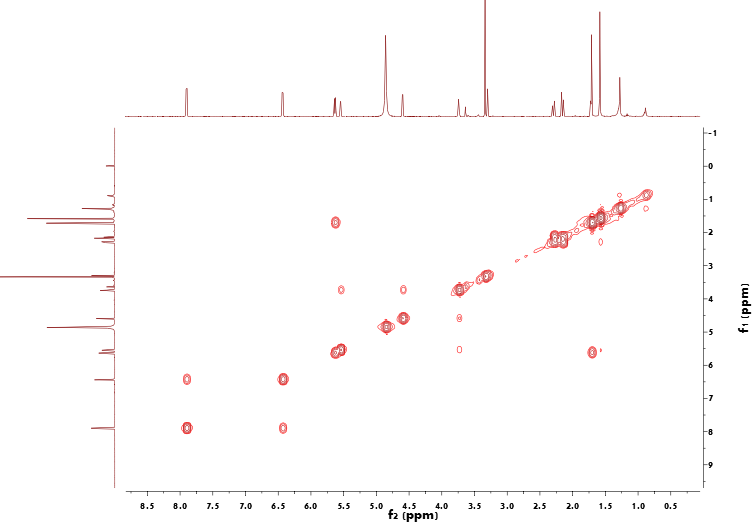


Figure S33. ^1^H-^1^H COSY spectrum of the compound **5**


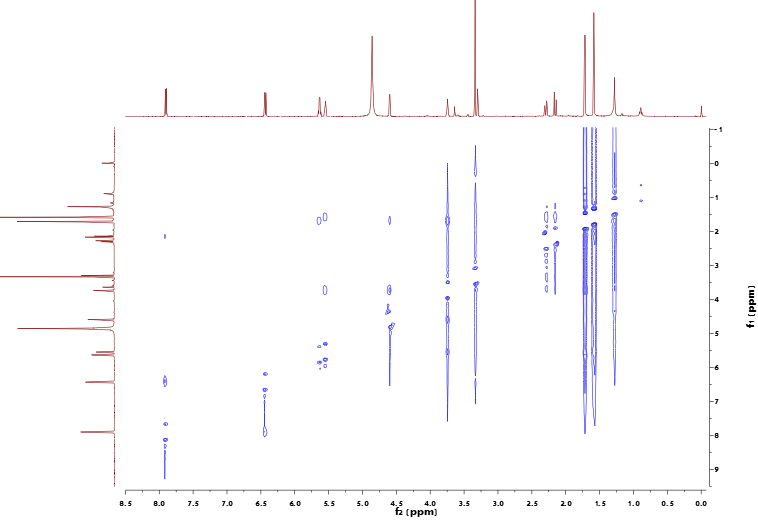


Figure S34. ROESY spectrum of the compound **5**


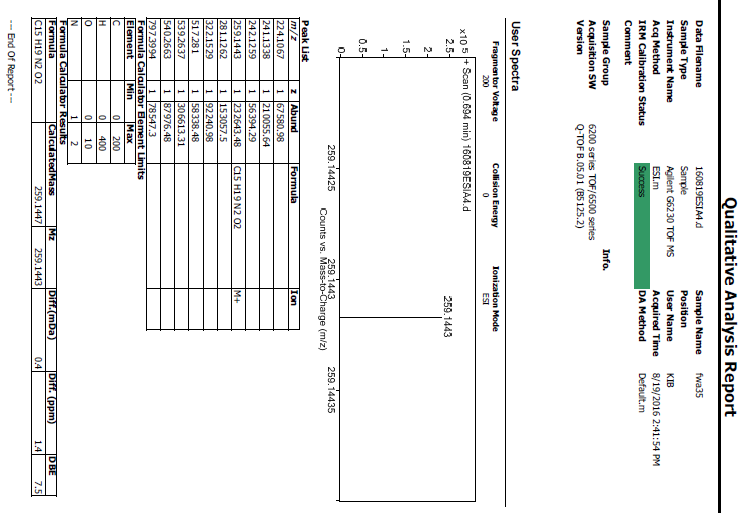


Figure S35. HR-ESI-MS spectrum of the compound **5**

The single-crystal X-ray diffraction of the compound **5**

Crystal data for mo_compound_**5**_0m: C_15_H_18_N_2_O_2_•H_2_O, *M* = 276.33, *a* = 14.227(7) Å, *b* = 12.474(6) Å, *c* = 8.948(5) Å, *α* = 90°, *β* = 113.406(7)°, *γ* = 90°, *V* = 1457.4(13) Å^3^, *T* = 100(2) K, space group *C*2, *Z* = 4, *μ*(MoKα) = 0.088 mm^-1^, 6582 reflections measured, 3687 independent reflections (*R_int_* = 0.0400). The final *R_1_* values were 0.0840 (*I* > 2*σ*(*I*)). The final *wR*(*F*^2^) values were 0.2254 (*I* > 2*σ*(*I*)). The final *R_1_* values were 0.0914 (all data). The final *wR*(*F*^2^) values were 0.2326 (all data). The goodness of fit on *F*^2^ was 1.168. Flack parameter = 0.3(7).

View of the molecules in an asymmetric unit.

Displacement ellipsoids are drawn at the 30% probability level.

iew of a molecule of compound_5 with the atom-labelling scheme.

Displacement ellipsoids are drawn at the 30% probability level.

View of the pack drawing of compound_**5**.

Hydrogen-bonds are shown as dashed lines.

Table 1. Crystal data and structure refinement for mo_ compound _**5**_0m.

Identification code mo_ compound _**5**_0m

Empirical formula C15 H20 N2 O3

Formula weight 276.33

Temperature 100(2) K

Wavelength 0.71073 Å

Crystal system Monoclinic

Space group C2

Unit cell dimensions a = 14.227(7) Å α= 90°.

b = 12.474(6) Å β= 113.406(7)°.

c = 8.948(5) Å γ = 90°.

Volume 1457.4(13) Å3

Z 4

Density (calculated) 1.259 Mg/m3

Absorption coefficient 0.088 mm-1

F(000) 592

Crystal size 0.630 x 0.380 x 0.280 mm3

Theta range for data collection 2.258 to 30.226°.

Index ranges -19<=h<=19, -17<=k<=16, -11<=l<=12

Reflections collected 6582

Independent reflections 3687 [R(int) = 0.0400]

Completeness to theta = 25.242° 98.3 %

Absorption correction Semi-empirical from equivalents

Refinement method Full-matrix least-squares on F2

Data / restraints / parameters 3687 / 1 / 184

Goodness-of-fit on F2 1.168

Final R indices [I>2sigma(I)] R1 = 0.0840, wR2 = 0.2254

R indices (all data) R1 = 0.0914, wR2 = 0.2326

Absolute structure parameter 0.3(7)

Extinction coefficient 0.047(8)

Largest diff. peak and hole 0.576 and -0.652 e.Å-3
